# The Philadelphia Theta Burst Project: A novel fMRI-guided electric field modeling dataset with reliability metrics

**DOI:** 10.1162/IMAG.a.1314

**Published:** 2026-10-05

**Authors:** David F. Gregory, Ivy Sun, Marta Teferi, Hannah Gura, Milan Patel, Abigail Casalvera, Sneha Chandrashekar, Kevin G. Lynch, Douglas H. Schultz, Ashish Jeyapratap, Quinn Tate, Walid Makhoul, Zhi-De Deng, Desmond J. Oathes, Yvette I. Sheline, Nicholas L. Balderston

**Affiliations:** Center for Neuromodulation in Depression and Stress, Department of Psychiatry, University of Pennsylvania, Philadelphia, PA, United States; Neuroscience Graduate Group, Perelman School of Medicine, University of Pennsylvania, Philadelphia, PA, United States; Department of Psychiatry, University of Pennsylvania, Philadelphia, PA, United States; Center for Brain, Biology and Behavior, University of Nebraska-Lincoln, Lincoln, NE, United States; Department of Psychology, University of Nebraska-Lincoln, Lincoln, NE, United States; Department of Physical Medicine and Rehabilitation, University of Pennsylvania, Philadelphia, PA, United States; Noninvasive Neuromodulation Unit, Experimental Therapeutics and Pathophysiology Branch, National Institute of Mental Health, National Institutes of Health, Bethesda, MD, United States; Center for Brain Imaging and Stimulation, Department of Psychiatry, University of Pennsylvania, Philadelphia, PA, United States; Penn Brain Science, Translation, Innovation, and Modulation Center, University of Pennsylvania, Philadelphia, PA, United States

**Keywords:** transcranial magnetic stimulation, theta burst stimulation, fMRI-guided targeting, electric field modeling, neuroimaging reliability, open dataset, working memory

## Abstract

Recent neuromodulation studies suggest that theta burst stimulation (TBS) applied to the dorsolateral prefrontal cortex (dlPFC) can influence neural circuits involved in cognitive control and emotion-related processing, with relevance for psychiatric disorders. However, large-scale datasets characterizing variability, reliability, and multimodal neural responses to TBS remain limited. To address this gap, we established the Philadelphia Theta Burst Project, a dataset of 100 healthy participants who completed 4 fMRI sessions following cumulatively increasing doses of intermittent or continuous TBS, with participants randomized to one protocol or the other. Each session includes multiband, multi-echo resting-state (16 minutes) and task-based fMRI (16 minutes) during a Sternberg working memory paradigm. TMS targeting was guided by task-evoked activation in the left dlPFC. Structural T1-weighted, T2-weighted, and diffusion-weighted imaging were also acquired. Here, we describe the dataset design, acquisition protocol, and data characteristics, and report initial measures of data quality and reliability. The completed dataset can be accessed at https://openneuro.org/datasets/ds008893, providing an open resource for future investigations of TBS mechanisms and individualized neuromodulation strategies.

## 1 Introduction

Transcranial magnetic stimulation (TMS) is a non-invasive neuromodulation technique that uses magnetic pulses to modulate neuronal activity in targeted brain regions. These pulses can alter functional connectivity, allowing researchers to causally probe the relationship between neural circuits and behavior (Bergmann & Hartwigsen, 2021; Hobot et al., 2021; Pascual-Leone, 2000). Clinically, TMS has demonstrated efficacy in treatment-resistant depression and is being explored for use in a range of neurological and psychiatric disorders, including anxiety, bipolar disorder, Alzheimer’s disease, Parkinson’s disease, multiple sclerosis, and stroke (Zhou et al., 2023). Despite its clinical success, the mechanistic underpinnings of TMS remain poorly understood, and its therapeutic potential may be limited by the lack of individualized targeting strategies (Fitzgerald et al., 2006; Lefaucheur et al., 2020). Most current applications use fixed stimulation sites, rather than tailoring targets based on functional neuroimaging. This limits precision and may reduce clinical impact (McClintock et al., 2018). Additionally, improved characterization of target regions for different symptom domains may clarify both the therapeutic and cognitive effects of TMS. Furthermore, the association between specific stimulation targets and various mental disorders remains unexplored, requiring more systematic investigation into the effects of stimulation on different brain regions (Rossi et al., 2021).

Recent studies have implicated the dorsolateral prefrontal cortex (dlPFC) in cognitive control (Webler et al., 2022) and emotion regulation (Joyce et al., 2025), offering insight into the potential mechanisms of action of dlPFC-targeted interventions (Miller & Cohen, 2001). TMS offers a promising approach for modulating dlPFC networks (Blumberger et al., 2018), but there is little consensus regarding the best approach to individualizing TMS targeted to the dlPFC (Cardenas et al., 2022; Jiang et al., 2025). Working memory tasks are well established (Sternberg, 1966), reliable (Forsyth et al., 2014; Kennedy et al., 2022), and able to robustly activate the dlPFC (Badre & Nee, 2018), which makes them well suited for use in individualized TMS-targeting algorithms (Balderston, Roberts, et al., 2020). Targeting based on working memory-related activation also has the advantage of including a positive manipulation check based on changes in behavioral performance (Teferi et al., 2023).

Studies have revealed changes in resting-state functional connectivity in response to TMS (Fox, Halko, et al., 2012). Mapping such differences with fMRI can better examine the relationship between different types of TMS and resting-state connectivity, thereby guiding more personalized treatment strategies. Among current TMS protocols, theta burst stimulation (TBS) is a high-efficiency protocol that mimics endogenous theta rhythms, modulating cortical excitability in minutes (Huang et al., 2005). To enhance targeting precision, electric field (E-field) modeling can estimate the spatial distribution of TMS-induced electric fields in the dlPFC, leveraging individual anatomical and functional data to optimize stimulation sites (Wu et al., 2025). This approach complements fMRI connectivity analyses, enabling circuit-specific interventions tailored to individual neural profiles (Balderston et al., 2024; Rajasekharan et al., 2025). The present dataset captures two forms of TBS—intermittent (iTBS) and continuous (cTBS)—administered in a repeated-measures design in a large sample, enabling future examination of effects on resting-state connectivity and working memory performance (Chung et al., 2019). Specifically, cTBS is often conceptualized as reducing cortical excitability and downregulating network activity, whereas iTBS is thought to enhance excitability and strengthen functional connections between prefrontal regions and subcortical targets (Suppa et al., 2008), though these directional effects remain an active area of investigation (Hermiller, 2024; Hussain & Freedberg, 2025). These opposing effects suggest differences in how the brain consolidates the stimulation-induced plasticity over time, which could be reflected in functional connectivity changes monitored by fMRI following iTBS and cTBS. However, we lack empirical data showing how or whether these protocols differentially affect functional connectivity.

While TBS protocols are increasingly used in both clinical and research settings, it remains unclear how different patterns affect large-scale brain connectivity (Eldaief et al., 2011). Critical questions persist regarding optimal dosage, differences in stimulation patterns, and timing of application both within and across sessions (Oberman et al., 2011). Additionally, the relationship between cognitive performance and stimulation patterns is not yet well understood (Hoy & Fitzgerald, 2010). Our laboratory established the Philadelphia Theta Burst Project to address these gaps by collecting a large-scale dataset of neuroimaging and behavioral measures following iTBS and cTBS in 200 healthy participants. In the current work, we describe the study design, data acquisition, and characteristics of the dataset, and report measures of data quality and reliability from the first 100 participants. The completed dataset can be accessed at https://openneuro.org/datasets/ds008893, as an open-access resource, including neuroimaging, behavioral, and stimulation-related data, providing a well-characterized resource for future research on TMS mechanisms and neuromodulation strategies. This dataset is intended to support future hypothesis-driven investigations of TBS-related changes in neural and behavioral measures and to facilitate development of individualized, circuit-informed neuromodulation approaches.

## 2 Materials and Methods

### 2.1 Participants

This study aims to recruit a total of 200 right-handed English-speaking participants aged 18 and 50 years from the Philadelphia, PA metropolitan area via flyers, advertisements, and listservs. The current report includes data from the first 100 participants enrolled. The exclusion criteria include non-English speaking; any significant medical problems; current or past Axis I psychiatric disorder(s) as determined by the Structured Clinical Interview for DSM-5 (SCID-5) (First, 2015); active or history of active suicidal ideation; alcohol or drug problems in the past year or lifetime alcohol or drug dependence; medications acting on the central nervous system; history of seizure; history of epilepsy or other neurological problems; increased risk of seizure for any reason; pregnancy; any medical condition that increases risk for fMRI or TMS; any metal in the body that would make having an MRI scan unsafe; any medical implants; and claustrophobia. The study protocol was approved by the institutional review board (IRB) for human subjects research at the University of Pennsylvania. Out of the 100 participants enrolled, 6 subjects did not meet the eligibility criteria during screening and were excluded from the study. A total of 87 participants completed the study (53 female, 31 male, 3 non-binary; mean age = 27.64 years, SD = 8.03). Six consented participants failed to meet the screening criteria. Seven consented participants were withdrawn from this current sample due to withdrawal from the study (two due to personal reasons; one due to dizziness and fainting after MRI scan; one due to MRI technical issues; two due to not being able to tolerate TMS; one no-show). All participants provided consent by signing an IRB-approved informed consent form. All procedures contributing to this study strictly comply with the ethical standards of the relevant national and institutional committees on human experimentation and with the Helsinki Declaration of 1975, as revised in 2008 (protocol #851028).

### 2.2 General procedures

Participants completed six visits over the course of 2 weeks (see Fig. 1). During the first week, participants completed a baseline visit and a baseline MRI session. The baseline visit included consent, eligibility screening, and questionnaires. The baseline MRI session was used for identifying the individualized TMS target (left dlPFC) with anatomical, resting-state, and task (Sternberg) scans. During the second week, participants completed three TMS visits across 4 consecutive days, each followed by a post-TMS MRI session approximately 24 hours later. Post-TMS MRI sessions included the same resting-state and Sternberg task scans as the baseline MRI session.

**Fig. 1.**
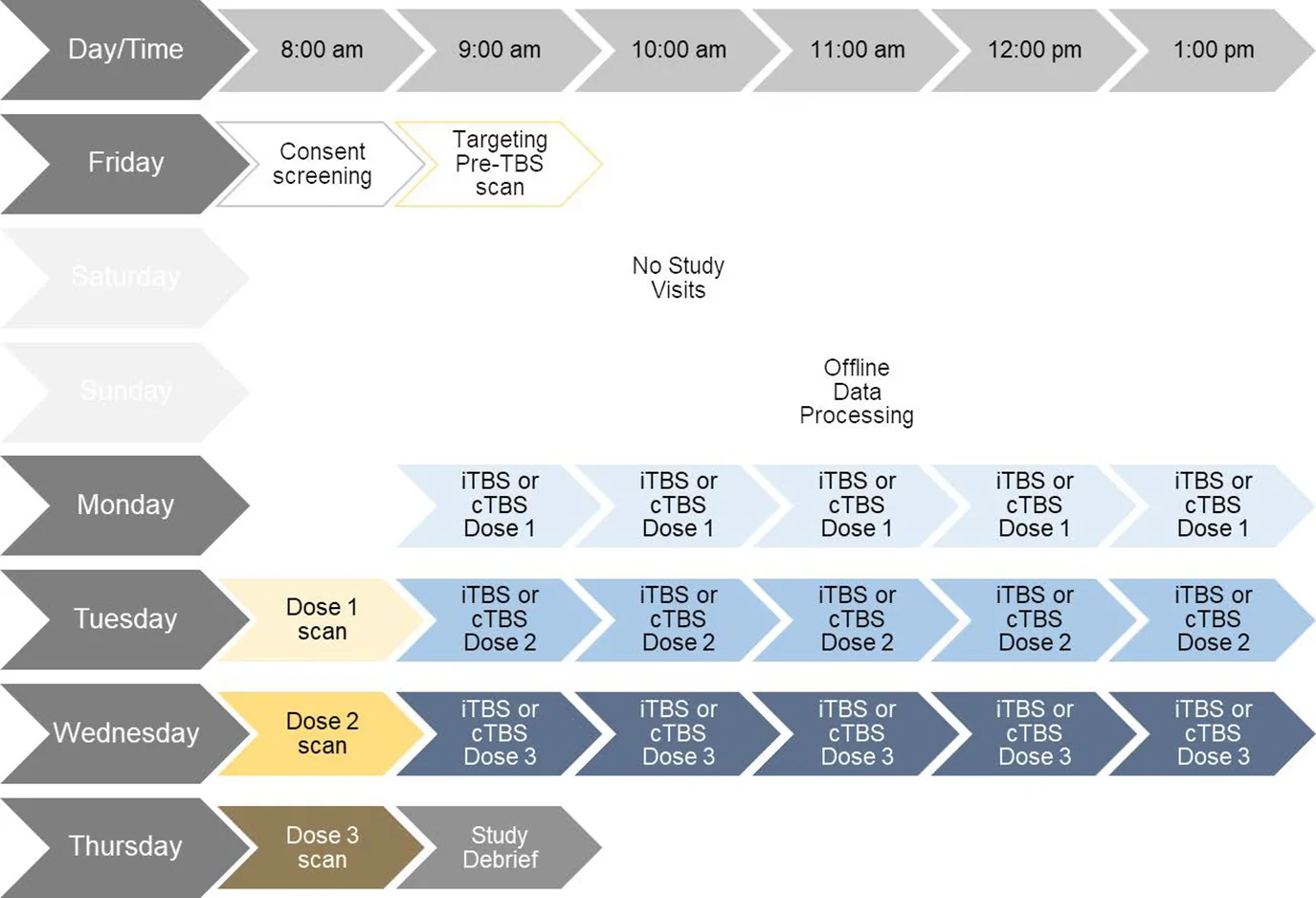
Study design and visit schedule. Timeline illustrating participant procedures across the study, beginning with the initial visit (consent, screening, and pre-TBS MRI targeting) and followed by three TMS dosing visits. On each visit, participants received five trains of either cTBS or iTBS separated by a 25-minute gap. Each train consisted of 600 pulses and delivered at 100% of motor threshold. Visits were then followed by a post-TMS MRI session 24 hours later. The study concluded with a study debrief. The example schedule is shown from Friday to the following Thursday.

### 2.3 Baseline visit procedure

All participants completed informed consent, MRI and TMS safety screening forms (CAMRIS and CNDS TASS), financial forms (W9 and C2), and a medical history questionnaire. The coordinator then administered the SCID (First, 2015), Montgomery–Asberg Depression Rating Scale (MADRS; Montgomery & Åsberg, 1979), and a protocol-specific eligibility checklist. Participants who met the eligibility criteria continued to complete a protocol-specific demographic questionnaire, the State/Trait Anxiety Inventory (STAI) (Spielberger, 2012), the Depression Anxiety Stress Scales (DASS) (Lovibond & Lovibond, 2011), and the Beck Anxiety Inventory (BAI) (Beck et al., 1988). Table 1 summarizes the questionnaire data. Table 2 summarizes measure reliability.

**Table 1.** Sample characteristics.

Measure	M	SD
Age	27.6	7.83
*Questionnaires*		
BAI	0.79	1.75
BDI	0.68	1.35
DASS_Depression	0.58	1.39
DASS_Anxiety	1	1.71
DASS_Stress	1.95	3.13
STAI_State	25.03	4.85
STAI_Trait	28.38	6.64
*Stimulation Intensity*		
B65	51.2	8.57
B65 A/P V/M	43.07	8.59

**Table 2.** Interclass correlations for Sternberg task.

Measure	ICC	F-value	df1	df2	p-value
*Accuracy*					
Maintain	0.62	5.92	81	162	4.77E-22
Sort	0.65	6.56	81	162	2.17E-24
Sort > Maintain	0.52	4.3	86	172	2.18E-16
*Reaction Time*					
Maintain	0.68	9.38	81	243	2.13E-42
Sort	0.77	14.57	81	243	2.40E-59
Sort > Maintain	0.48	4.66	86	243	5.61E-22
*dlPFC BOLD*					
Maintain	0.54	5.76	86	258	4.57E-28
Sort	0.62	7.56	86	258	5.02E-37
Sort > Maintain	0.41	3.75	86	258	1.67E-16

### 2.4 TMS visit procedure

During the TMS visit, participants wore a headband with neuronavigation tracking sensors. The participant’s head was registered to the MRI scan in Brainsight using standard fiducial landmarks (nasion and bilateral tragi). On the first TMS visit, motor threshold was determined using a parameter estimation by sequential testing (PEST) procedure (Borckardt et al., 2006). After obtaining the motor threshold, the coil was positioned over the left dlPFC to deliver either iTBS or cTBS. On each visit, participants received five trains of either cTBS or iTBS separated by a 25-minute gap. Each train was 600 pulses and delivered at 100% of motor threshold. Participants rated perceived stimulation intensity on a 1–10 scale after each stimulation run, and stimulation intensity was adjusted within protocol limits based on participant comfort ratings.

### 2.5 Scan visit procedure

During the MRI sessions, participants were escorted to the scanner and screened using a handheld metal detector prior to entering the MRI suite. Participants were provided with earplugs, a response button box, an emergency squeeze ball, and foam padding to minimize head motion. For the baseline MRI session, structural scans were acquired along with two resting-state runs and two Sternberg working memory (WM) task runs. For post-TMS MRI sessions, participants completed two resting-state runs and two Sternberg WM task runs, with brief breaks between runs. The diffusion-weighted scan was collected during the second or third scan block, depending on time constraints.

### 2.6 Sternberg working memory task

During the baseline MRI session, participants completed two runs of the Sternberg WM task during fMRI acquisition (see Fig. 2). There were no threat blocks and no shock during this baseline MRI session. The task included 12 trials each for the sort and maintain conditions. Sort trials required participants to reorder the presented letters into alphabetical order prior to recall, whereas maintain trials required participants to hold the original letter sequence in memory and reproduce it in the same order. The letter presentation (1.5–2.5 seconds), retention interval (6.5–8.5 seconds), and intertrial interval (5–8 seconds) were jittered across trials, whereas instruction (1 second) and response prompt (3 seconds) durations were fixed.

**Fig. 2.**
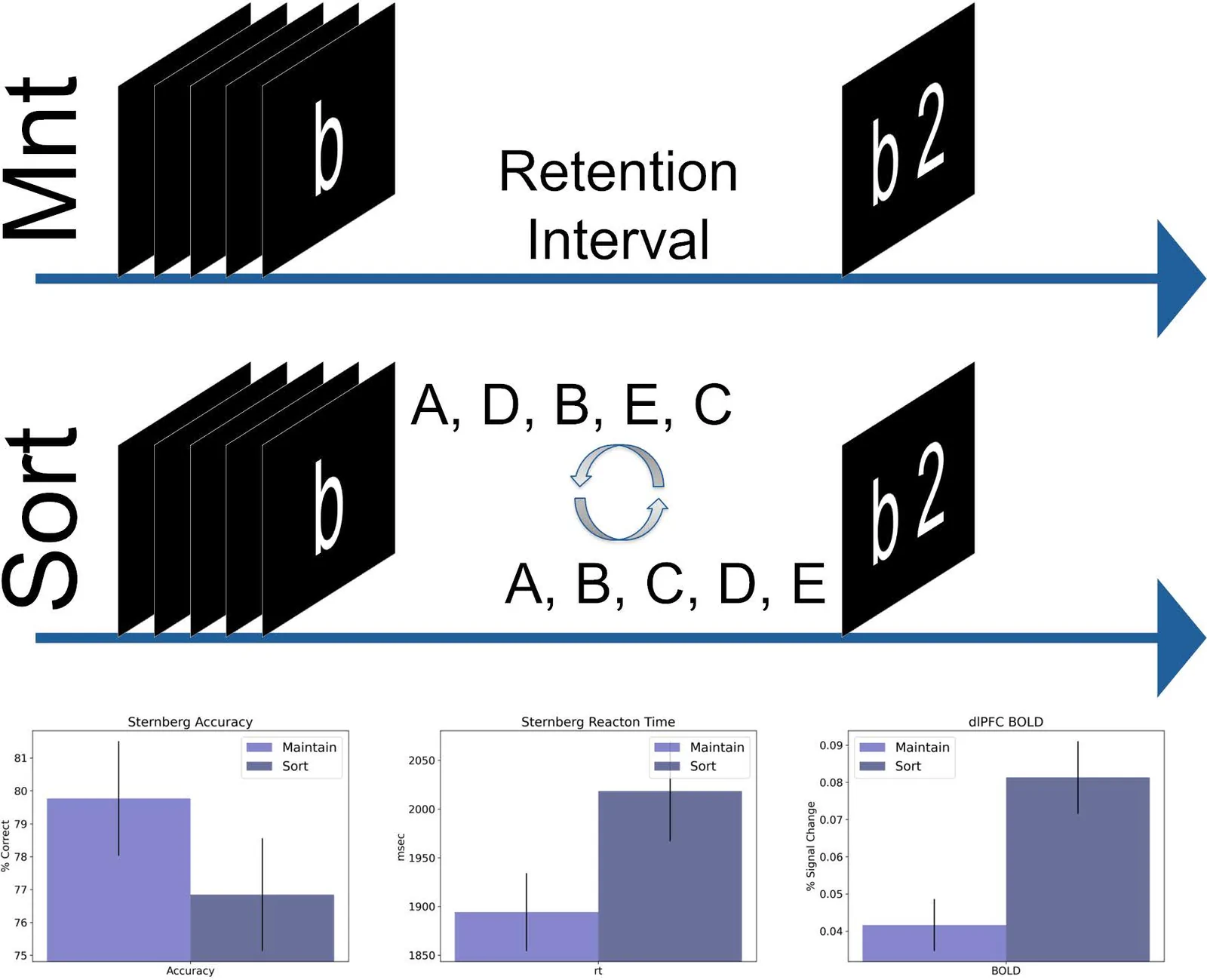
Sternberg working memory task. Schematic of the Sternberg working memory task illustrating maintain (Mnt) and sort conditions. Participants encode a sequence of letter stimuli and maintain the order across a delay period (Mnt condition) or mentally reorder the sequence alphabetically during the delay (Sort condition). A probe item is then presented, and participants indicate whether it matches the encoded (or manipulated, in the Sort condition) sequence. Below the Sternberg schematic, (Left) Accuracy during maintain and sort conditions. (Middle) Reaction time during maintain and sort conditions. (Right) dlPFC BOLD percent signal change during maintain and sort conditions.

### 2.7 Scanning protocol

MRI data were acquired using a 3 Tesla Siemens Prisma scanner with a 64-channel head coil (Erlangen, Germany). Structural scans included a T1-weighted (T1w) MPRAGE (TR = 2200 ms; TE = 4.67 ms; flip angle = 8°) with 160, 1 mm axial slices (matrix = 256 × 256; field of view (FOV) = 240 × 240 mm), and a T2-weighted (T2w) image (TR = 3200 ms; TE = 563 ms; flip angle = variable) with 160, 1 mm axial slices (256 × 256; FOV = 240 × 240 mm). Diffusion-weighted images were acquired using a single-shot spin-echo echo-planar imaging (EPI) sequence with multi-band acceleration (factor = 4). Acquisition parameters included 1.5 mm isotropic voxels, TR = 3230 ms, TE = 89.2 ms, FOV = 210 × 210 mm, 96 contiguous axial slices, and a multi-shell protocol with 45 non-collinear diffusion directions at b = 1500 s/mm², 45 non-collinear diffusion directions at b = 3000 s/mm², and 8 non-diffusion-weighted (b0) volumes. Reverse-phase encoded b0 images (anterior–posterior and posterior–anterior) were collected for susceptibility distortion correction. Functional data for resting-state and task runs consisted of four runs per session, each containing 450 whole-brain BOLD volumes (TR = 1000 ms; multi-echo TEs = 13.8, 37.65, 61.5 ms; flip angle = 40°; multiband factor = 6), acquired using 66 axial slices (2 mm isotropic voxels; matrix = 110 × 110; FOV = 220 × 220 mm) aligned to the AC–PC line.

### 2.8 fMRI preprocessing

fMRI data were processed using afni_proc.py (AFNI; Cox, 1996). The following preprocessing blocks were used: despike, tshift, align, volreg, blur, mask, scale, regress. During preprocessing, (1) the images were despiked and slice time corrected, (2) images were aligned to the T1w data using a Local Pearson Correlation cost function, (3) individual volumes were registered to the image with the fewest outliers, (4) multi-echo data were optimally combined using *tedana* (AFNI; DuPre et al., 2021), (5) images were blurred with a 2 mm Gaussian kernel, (6) data were masked using a union of the EPI-derived brain mask and the skull-stripped T1w image, (7) they were then scaled so the mean of each voxel time series was 100. The first four TRs and volumes with frame-wise displacement >0.5 mm or >15% outlier voxels were censored prior to GLM estimation. For resting-state data, the first-level GLM included nuisance regressors for the six motion parameters and their temporal derivatives, polynomial terms modeling baseline drift, white matter signal, CSF signal, and global signal. For task data, events during the WM task were modeled using variable-duration block regressors to account for jittered event timing.

### 2.9 Electric field modeling and coil optimization

The SimNIBS software package (version 2.1) was used to create finite element models representing the head and coil geometries from the T1w and T2w scans (Thielscher et al., 2015). Images were segmented into tissue compartments (scalp, skull, CSF, gray matter, and white matter) and meshed using Gmsh routines (Thielscher et al., 2011). For the E-field models, the roll and pitch of the coil were set perpendicular to the cortical surface at the stimulation site. Twenty-four E-field models were computed at evenly spaced yaw orientations (15° increments) centered on the scalp target. E-field magnitude was averaged within the left dlPFC region of interest, and the yaw orientation corresponding to the maximal E-field within this region of interest was selected as the stimulation orientation (Balderston, Beydler, et al., 2020; Balderston, Roberts, et al., 2020).

### 2.10 Target localization

Sternberg WM task data were used to derive individualized target coordinates for each participant (see Fig. 3; Balderston, Beydler, et al., 2020; Teferi et al., 2023). BOLD activity during the retention interval was masked using a functional region of interest encompassing the left dlPFC, defined from a group-level analysis of a previously collected Sternberg WM dataset (Balderston, Flook, et al., 2020; Balderston, Roberts, et al., 2020). Activity was contrasted between sort and maintain trials, and the peak voxel within this mask was identified as the stimulation target. Target coordinates were projected onto the scalp mesh using a nearest-neighbor search.

**Fig. 3.**
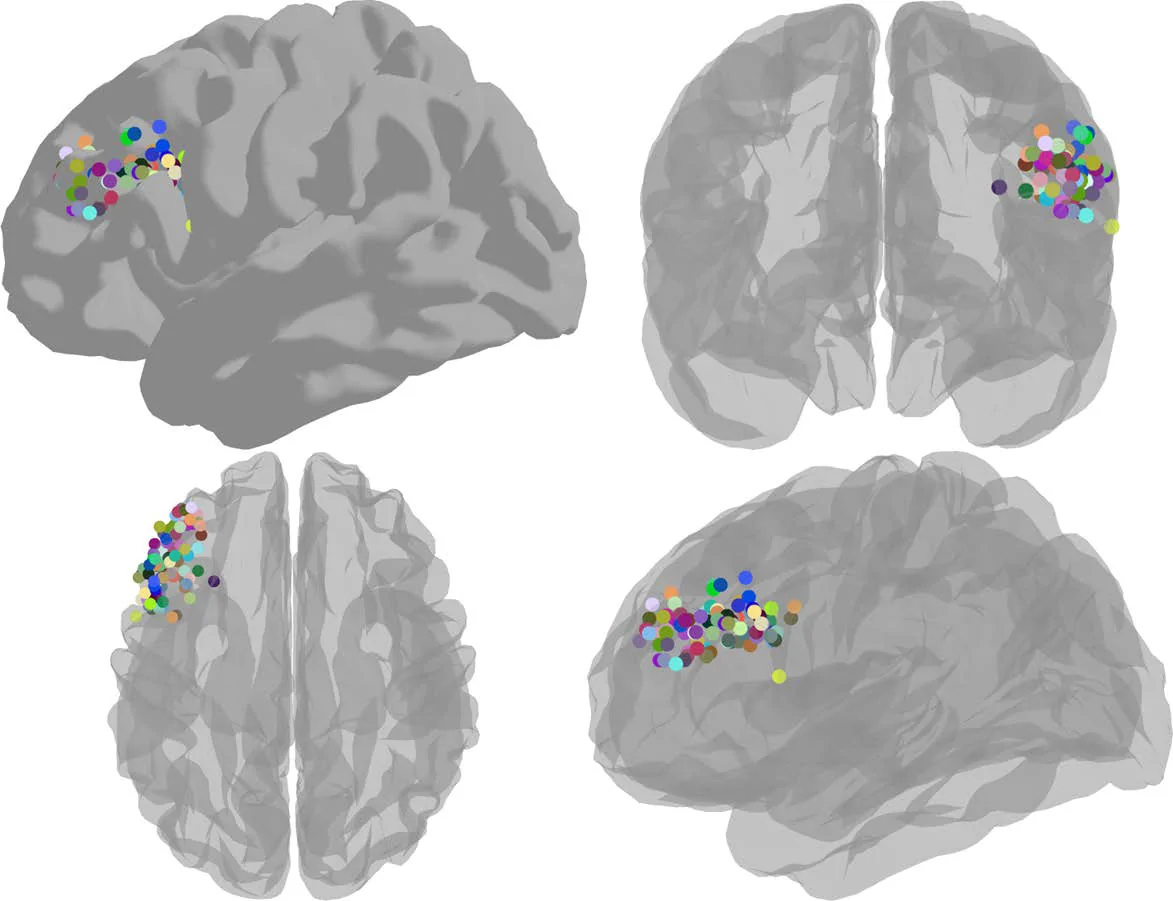
Transcranial magnetic stimulation (TMS) sites. Representative left dlPFC theta burst stimulation (TBS) target locations shown across anatomical planes, including sagittal (top left), coronal (top right), axial (bottom left), and subcortical sagittal views (bottom right).

### 2.11 Active stimulation

A Magventure MagPro X100 stimulator with a B65 figure-8 coil was used for the TBS sessions.

### 2.12 iTBS parameters

During each iTBS session, intermittent TBS was delivered at 100% of resting motor threshold (RMT) in 2 seconds trains consisting of bursts of 3 pulses at 50 Hz, repeated at 5 Hz, with 8 seconds inter-train intervals, for a total of 600 pulses per session.

### 2.13 cTBS parameters

During each cTBS session, continuous TBS was delivered at 100% of RMT consisting of bursts of 3 pulses at 50 Hz repeated at 5 Hz for 40 seconds, for a total of 600 pulses per session.

### 2.14 Neuronavigation

We used a frameless stereotaxic neuronavigation system (Brainsight; Rogue Research Inc., Montreal, Canada) to guide coil placement. Subject-specific NIfTI data were aligned to each participant’s native T1w image for visualization of the stimulation target and coil orientation. Scalp and cortical surfaces were reconstructed from the T1w image. Prior to stimulation, each participant’s head was co-registered to their T1w using fiducial landmarks at the nasion and tragi, followed by 50–100 additional scalp surface points for refinement. The predefined target and orientation were used in Brainsight to generate a coil positioning trajectory. TMS was delivered according to this trajectory, and neuronavigation accuracy was quantified using Brainsight estimates of scalp-to-cortex distance, positional error, and angular error (roll/pitch/yaw).

## 3 Results

### 3.1 Stimulation sites

Subject-specific TMS targets were defined as 6 mm radius spheres centered on peak BOLD activity during the Sternberg working memory (WM) task. These peaks were constrained to dorsolateral prefrontal cortex (dlPFC) voxels within a predefined functional mask and identified independently for each participant (see Fig. 3).

### 3.2 Neuronavigation accuracy

Across participants, the mean scalp-to-cortex distance at the stimulation site was 26.4 mm (SD = 7.8). Mean distance error was 3.6 mm (SD = 5.2), with angular errors of 1.9° (SD = 2.3) for roll/pitch and 8.5° (SD = 12.9) for yaw.

### 3.3 Sternberg accuracy

We compared accuracy between maintain and sort conditions using a paired t-test. Accuracy was significantly higher in the maintain condition (M = 79.7%) than in the sort condition (M = 76.8%), t(86) = 2.84, p = 0.006, d = 0.31 (Fig. 2).

### 3.4 Sternberg reaction time

We compared reaction times between maintain and sort conditions using a paired t-test. Participants responded faster in the maintain condition (M = 1890 ms) than in the sort condition (M = 2200 ms), t(86) = -4.36, p < 0.001, d = -0.47 (Fig. 2).

### 3.5 Sternberg dlPFC BOLD

We examined dlPFC BOLD signal differences between sort and maintain conditions. The sort condition (M = 0.08% change) evoked significantly greater dlPFC activation than the maintain condition (M = 0.04% change), t(86) = -6.96, p < 0.001, d = -0.75 (Fig. 2).

### 3.6 Sternberg whole-brain BOLD

This analysis examined voxel-wise whole-brain BOLD activity across encoding, retention, and response intervals. During the retention interval, increased BOLD activity was observed in frontal and parietal regions, alongside decreased BOLD signal in regions commonly associated with the default mode network, including ventromedial prefrontal cortex, posterior cingulate cortex, and hippocampus. dlPFC activity during the retention interval was consistently observed across contrasts and was consistent with ROI-based analyses (Fig. 4).

**Fig. 4.**
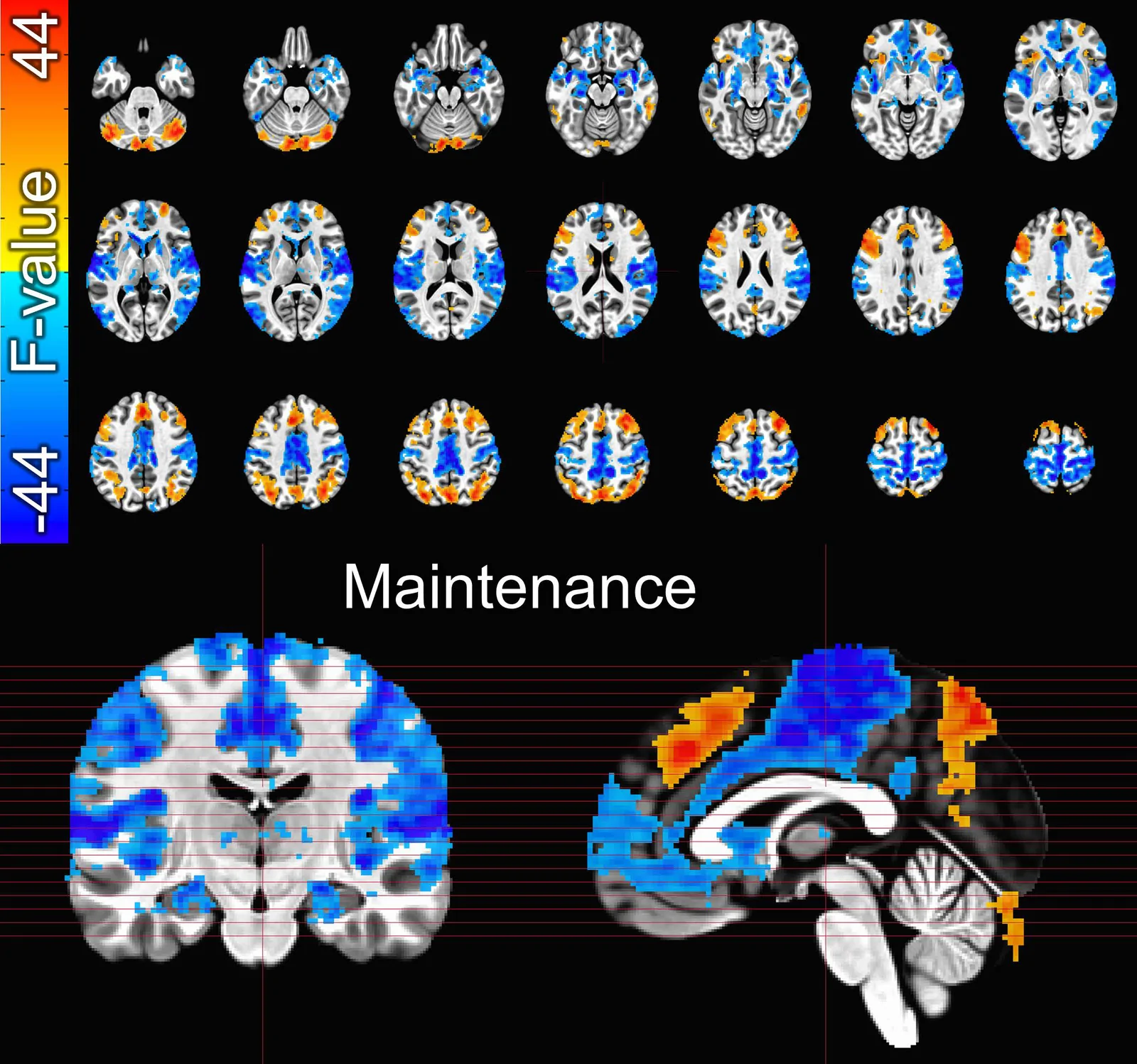
Brain activation during working memory task. Whole-brain F-statistic maps illustrating task-related effects during the working memory task, shown for the maintain condition (axial slices). Coronal and sagittal views depict the spatial distribution of F-statistics across the brain.

### 3.7 Average resting-state across sessions

Resting-state functional connectivity (rsFC) was estimated as Pearson correlation coefficients between regional time series and averaged across subjects for each pair of regions in the 333-region Gordon atlas (Fig. 5). The resulting connectivity pattern was consistent with previous reports using this atlas (Gordon et al., 2016).

**Fig. 5.**
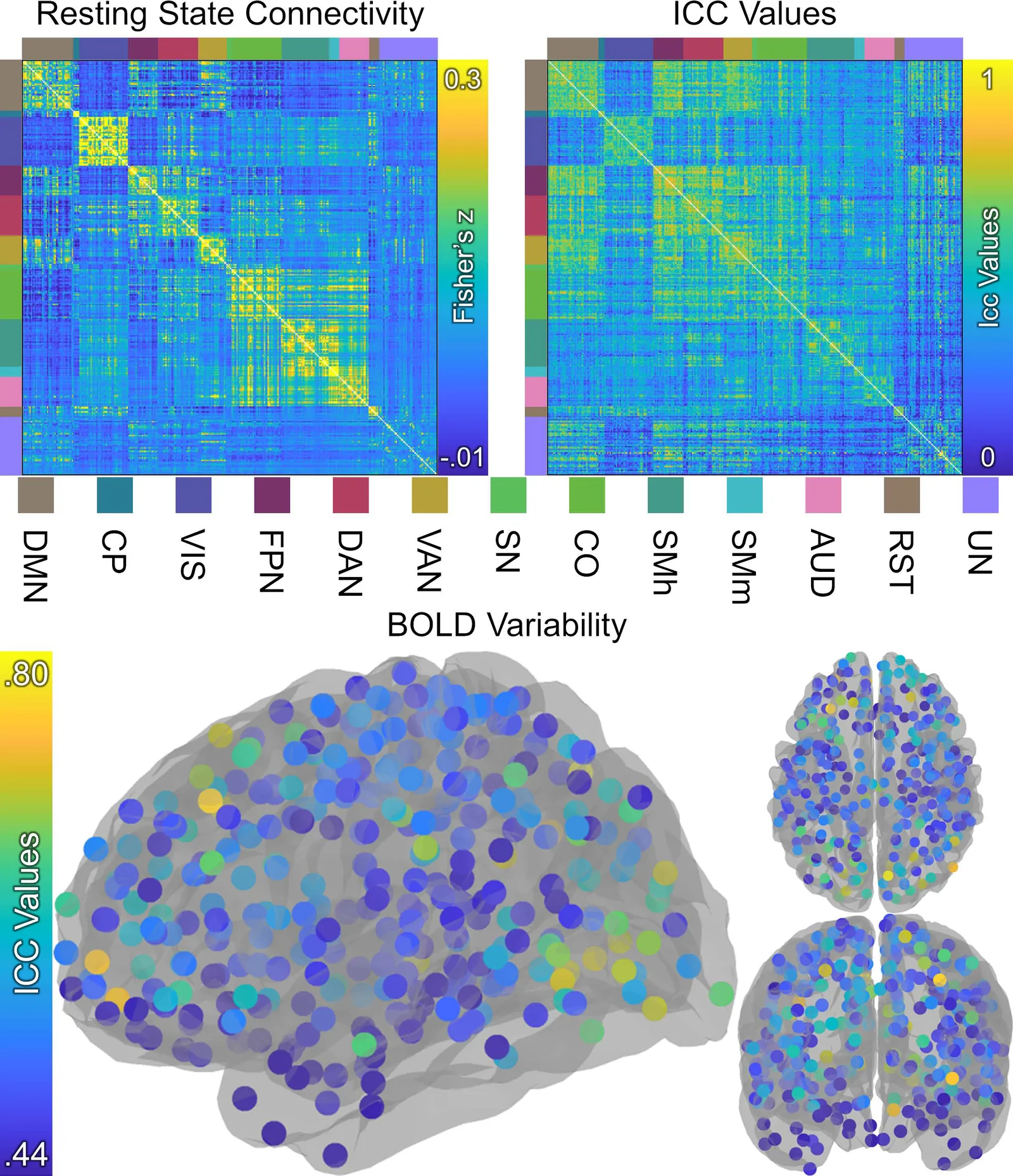
Brain network correlation connectivity and BOLD variability reliability. Top schematic showing connectivity and correlations of brain networks; (Left) resting-state functional connectivity matrix showing Pearson correlation coefficients between major brain networks (range: −0.1 to 0.3). (Right) Intraclass correlation coefficient (ICC) matrix showing test–retest reliability of connectivity values (range: 0 to 1). Below the correlation connectivity figures are the BOLD reliability; (Left) resting-state functional connectivity matrix showing Pearson correlation coefficients between major brain networks (range: −0.1 to 0.3). (Right) Intraclass correlation coefficient (ICC) matrix showing test–retest reliability of connectivity values (range: 0 to 1). Networks include default mode (DMN), cingulo-opercular (CO), cingulo-parietal (CP), hand sensorimotor (SMh), visual (VIS), mouth sensorimotor (SMm), frontoparietal (FPN), auditory (AUD), dorsal attention (DAN), retrosplenial-temporal (RST), ventral attention (VAN), unassigned (UN), and salience (SN) networks.

### 3.8 Average BOLD signal variability across sessions

BOLD variability was computed as the mean square of successive differences in the preprocessed resting-state time series and averaged across subjects for each region in the 333-region Gordon atlas (Garrett et al., 2011; Nomi et al., 2017; Von Neumann et al., 1941). BOLD variability did not show consistent differences across networks or sessions (see Table 3 for mean variability values across networks and sessions).

**Table 3.** BOLD variability across sessions and networks.

Network	Session 1	Session 2	Session 3	Session 4
Default mode	0.101 (0.025)	0.099 (0.025)	0.101 (0.029)	0.099 (0.024)
Cingulo-opercular	0.147 (0.033)	0.146 (0.033)	0.147 (0.037)	0.146 (0.037)
Cingulo-parietal	0.163 (0.044)	0.160 (0.044)	0.163 (0.044)	0.157 (0.042)
Hand sensorimotor	0.128 (0.045)	0.125 (0.041)	0.128 (0.045)	0.124 (0.044)
Visual	0.133 (0.035)	0.131 (0.031)	0.131 (0.036)	0.130 (0.038)
Mouth sensorimotor	0.083 (0.025)	0.083 (0.025)	0.084 (0.028)	0.081 (0.025)
Frontoparietal	0.114 (0.032)	0.113 (0.034)	0.115 (0.037)	0.112 (0.032)
Auditory	0.234 (0.063)	0.234 (0.060)	0.234 (0.066)	0.232 (0.064)
Dorsal attention	0.119 (0.033)	0.117 (0.033)	0.119 (0.036)	0.116 (0.033)
Retrosplenial-temporal	0.289 (0.078)	0.285 (0.074)	0.285 (0.079)	0.283 (0.082)
Ventral attention	0.163 (0.036)	0.163 (0.036)	0.164 (0.042)	0.162 (0.040)
Unassigned	0.198 (0.055)	0.198 (0.057)	0.200 (0.061)	0.198 (0.061)
Salience	0.252 (0.074)	0.246 (0.062)	0.250 (0.082)	0.245 (0.059)

### 3.9 ICC values

We computed intraclass correlation coefficients (ICCs) to assess test–retest reliability across visits. ICCs were computed separately for accuracy, reaction time, dlPFC BOLD activity, and rsFC. Accuracy and reaction time were averaged across trials within condition and session. dlPFC BOLD activity was extracted from the functionally defined ROI and averaged across task blocks. Resting-state connectivity was computed as Fisher z-transformed correlations between each pair of regions in the network parcellations (Fig. 5). ICCs were categorized as <0.50 (poor), 0.50–0.75 (moderate), 0.75–0.90 (good), and >0.90 (excellent). ICCs for reaction time and dlPFC BOLD in the sort versus maintain contrast were below 0.50, whereas other measures fell within the moderate-to-good range (Table 2). The poor ICC values observed for dlPFC BOLD activity (Sort > Maintain; ICC = 0.41) and reaction time (ICC = 0.48) warrant interpretation. Several factors likely contribute to these modest estimates, including state-dependent variability in prefrontal engagement across sessions, subtle differences in task strategy across visits, and known reliability constraints of single-region BOLD contrasts in prefrontal cortex during working memory paradigms (Ten Hove et al., 2024; Zuo et al., 2019). Modest baseline reliability in task-evoked dlPFC engagement constrains sensitivity for detecting stimulation-induced changes against this background variability, and individual-differences analyses linking TBS-induced network changes to behavioral outcomes should account for this limitation when interpreting effect sizes and statistical power. rsFC ICCs showed patterns broadly similar to previous reports from the Human Connectome Project, with within-network connections generally higher than between-network connections (Tozzi et al., 2020). ICCs for BOLD variability are plotted in Figure 5, with most regions falling in the moderate-to-good range, consistent with prior test–retest estimates (Wehrheim et al., 2024).

## 4 Discussion

As TMS is increasingly used in the treatment of neurological and psychiatric disorders, characterizing the neural and behavioral landscape of theta burst stimulation (TBS) responses at scale becomes critical (Cole et al., 2016; Fox, Halko, et al., 2012). The Philadelphia Theta Burst Project was designed to capture these measures systematically, collecting neuroimaging and behavioral data following two patterns of TBS to the left dorsolateral prefrontal cortex (dlPFC)—intermittent (iTBS) and continuous (cTBS)—across repeated measures in a large sample. Once completed, this dataset will represent one of the largest openly available collections of neuroimaging and behavioral data following TBS, offering opportunities to refine individualized TMS protocols and extend applications in both research and clinical contexts (Balderston, Roberts, et al., 2020).

Beyond connectivity outcomes, the dataset includes working memory (WM) as a sensitive behavioral assay for future examination of TMS-induced modulation. WM performance is responsive to dlPFC stimulation (Balderston, Flook, et al., 2020), providing a well-validated measure that future analyses can use to link neural changes to cognition. Similarly, rs-FC offers a reproducible index of network-level effects of TBS (Fox, Buckner, et al., 2012; Noble et al., 2019), complementing task-based measures by capturing broader systems-level dynamics. Measures of BOLD variability have been associated with developmental changes through the lifespan (Garrett et al., 2011; Kielar et al., 2016) and several clinical populations (Easson & McIntosh, 2019; Nomi et al., 2017; Scarapicchia et al., 2018). Physiological changes in vasculature, neural activity, and neurovascular coupling (Amen et al., 2016; Park et al., 2009; Soustiel & Sviri, 2007; Ueda et al., 2003) are also reflected in measures of BOLD variability. Variability measures complement connectivity results and may provide additional context for interpreting network-level changes. The co-acquisition of these measures within the same dataset positions future analyses to examine their relationship with cognitive outcomes. Repeated measurement of both WM and rs-FC across incremental TBS doses provides the structure needed to test dose-dependent hypotheses in future work, supporting development of individualized TMS strategies.

The data included in this dataset are comparable with existing work. For the Sternberg task, accuracy and reaction time outcomes are within the range of previously published work and BOLD patterns match as well (Balderston, Roberts, et al., 2020; Balderston et al., 2016; Sternberg, 1966; Teferi et al., 2024). The reliability of resting-state functional connections matches what was seen in the human connectome project (Tozzi et al., 2020), despite the difference in scan duration (1 hour [HCP] vs. 16 minutes [current study]). BOLD variability across networks is consistent with work using the MSSD method (Anderson et al., 2023; Easson & McIntosh, 2019; Mohr & Nagel, 2010; Zhang et al., 2022), and did not vary across sessions.

Characterizing how TBS modulates functional networks at scale is essential for developing computational models for precision targeting (Cole et al., 2016; Sun et al., 2024). Prior work {Citation}implemented an E-field/connectivity mapping approach to predict rs-FC changes following iTBS, showing correlations between alterations in connectivity and the spatial distribution of the E-field (Balderston et al., 2022). In subsequent work, a prospective targeting algorithm was developed to individualize TMS sites to networks associated with depression symptoms (Cash, Cocchi, et al., 2021; Weigand et al., 2018). The current dataset is designed to support future mechanistic investigations of TBS-induced network changes, with the potential to strengthen predictive modeling and inform connectivity-guided interventions. Clinical studies support this approach, demonstrating that iTBS-induced rs-FC changes correspond systematically to E-field topography (Balderston et al., 2024; Cash, Weigand, et al., 2021; Weigand et al., 2018). These findings lay the foundation for translating mechanistic models into precision neuromodulation protocols.

Connectivity-guided TMS builds on prior work demonstrating the relevance of subgenual and prefrontal networks in predicting treatment response. Individualized targeting based on functional connectivity has improved outcomes in depression and other neuropsychiatric conditions (Siddiqi et al., 2021; Weigand et al., 2018). Reproducibility and reliability of these targets have been confirmed in multiple studies, supporting precision neuromodulation applications in research and clinical applications (Balderston et al., 2024; Zhou et al., 2023).

Optimizing connectivity-guided TMS requires assessment of reliability across multiple scans. The dataset, with 4 scans per participant across 200 individuals, represents a robust resource (Glick et al., 2025). Factors affecting connectivity consistency include single- versus multi-echo fMRI sequences, with multi-echo showing improved reliability via reduced dropout and noise (Kundu et al., 2013; Lynch et al., 2020). Functional mask size influences stability of connectivity estimates (Smith et al., 2013), and selection of normative network seeds affects connectivity profiles, emphasizing standardized protocols (Cole et al., 2016). Dynamic fluctuations in connectivity may also shape TMS outcomes, highlighting the need to consider temporal variability in targeting (Hutchison et al., 2013). These factors represent understudied areas for future exploration using this dataset.

Large-scale TMS and TBS studies highlight both potential and the limitations of targeting strategies across clinical and mechanistic populations. Clinical trials, including THREE-D, showed non-inferiority of iTBS versus conventional 10 Hz stimulation using standard targeting, while individualized connectivity-guided protocols show advantages in treatment-resistant samples (Blumberger et al., 2018; Morriss et al., 2024). Large-N clinical datasets (Fitzgerald et al., 2009; Zhao et al., 2024) demonstrate that targeting strategy shapes response magnitude and reproducibility. Mechanistic studies in healthy participants indicate that MRI-guided neuronavigation ensures anatomical precision (Vékony et al., 2018), but group-level targeting may obscure individual variability (Xu et al., 2024). Recent TMS-fMRI datasets across 150+ individuals (Glick et al., 2025) illustrate the translational potential of combining stimulation with neural measures while revealing methodological heterogeneity. The current dataset integrates individualized stimulation with systematic behavioral and network-level outcomes, positioning future analyses to bridge mechanistic insights and clinical translation.

Dataset strengths include large sample size with repeated scans enabling reliability assessment, comparison of iTBS and cTBS in a repeated-measures design, and integration of task-based and rs-FC measures. Limitations include the exclusive healthy adult sample, restricting generalizability, reliance on a single cognitive task domain, and untested longer-term effects of repeated TBS. Additionally, primary neuroimaging analyses focus on the dlPFC as an a priori region of interest, which limits what can be inferred about TBS’s network-wide effects. TBS is known to influence distributed networks, and future analyses examining action-related networks—including the SCAN network (Chen et al., 2025)—are well motivated by the whole-brain acquisition structure of this dataset. Hypothesis-driven investigations of distributed network responses are planned for future work, including an in-progress Sternberg task study examining working memory and subcortical whole-brain analyses. Residual network variability may also affect targeting precision (Lynch et al., 2020; Noble et al., 2019).

In summary, this paper describes the Philadelphia Theta Burst Project dataset, a large, multi-scan collection of neuroimaging and behavioral data following iTBS and cTBS to the dlPFC. The dataset combines individualized connectivity-guided targeting, repeated measures, and both resting-state and task-based fMRI, providing a well-characterized resource for probing TMS mechanisms in future work. Initial quality assurance and reliability metrics reported here support the dataset’s suitability for future investigations of precision neuromodulation and clinical protocol optimization. The resulting open-access dataset offers a resource for replication, secondary analyses, and development of individualized, circuit-informed TMS interventions (Balderston et al., 2024; Glick et al., 2025). A de-identified derivative dataset will also be deposited on OpenNeuro to ensure barrier-free global access.

## Data Availability

Data used in this study can be accessed using the weblink: https://openneuro.org/datasets/ds008893 All code used to preprocess these data can be found at https://github.com/balders2/ptbsc.
